# Photodynamic therapy – hypericin tetraether liposome conjugates and their antitumor and antiangiogenic activity

**DOI:** 10.1080/10717544.2018.1531954

**Published:** 2019-01-28

**Authors:** Nikola Plenagl, Lili Duse, Benjamin Sebastian Seitz, Nathalie Goergen, Shashank Reddy Pinnapireddy, Jarmila Jedelska, Jana Brüßler, Udo Bakowsky

**Affiliations:** Department of Pharmaceutics and Biopharmaceutics, University of Marburg, Marburg, Germany

**Keywords:** Antitumor, antiangiogenic, photodynamic therapy, hypericin, liposomes

## Abstract

Photodynamic therapy (PDT) is an established noninvasive tumor treatment. The hydrophobic natural occurring pigment hypericin shows a lot of attractive properties for the application in PDT. Hence, the administration to biological systems or patients requires the formulation in drug carriers enabling sufficient bioavailability. Therefore, free hypericin was encapsulated by the thin film hydration method or a hypericin-hydroxypropyl-β-cyclodextrin inclusion complex (Hyp-HPβCD) was incorporated by dehydration-rehydration vesicle method in either conventional or ultra-stable tetraether lipid (TEL) liposomes. The hydrodynamic diameter of the prepared nanoformulations ranged between 127 and 212 nm. These results were confirmed by atomic force microscopy. All liposomes showed a good stability under physiological conditions. TEL liposomes which tend to build more rigid bilayers, generate higher encapsulation efficiencies than their conventional counterparts. Furthermore, the suitability for intravenous application was confirmed by hemocompatibility studies resulting in a hemolytic potential less than 20% and a coagulation time less than 50 sec. The uptake of liposomal hypericin into human ovarian carcinoma cells (SK-OV-3) was confirmed using confocal microscopy and further characterized by pathway studies. It was demonstrated that the lipid composition and intraliposomal hypericin localization influenced the anti-vascular effect in the chorioallantoic membrane (CAM). While hypericin TEL liposomes exhibit substantial destruction of the microvasculature drug-in-cyclodextrin TEL liposomes showed no effect. Nevertheless, both formulations yielded severe photocytotoxicity in SK-OV-3 cells in a therapeutic dosage range. Conclusively, hypericin TEL liposomes would be perfectly suited for anti-vascular targeting while Hyp-HPβCD TEL liposomes could deliver the photosensitizer to the tumor site in a more protected manner.

## Introduction

A combination of three nontoxic elements is utilized to treat oncological diseases in the so-called photodynamic therapy (PDT). A photosensitizer (PS) which is administered locally or systemically to a patient is irradiated with light of an appropriate wavelength in the presence of molecular oxygen. Thereby reactive oxygen species (ROS) are generated, which cause oxidative damage to proteins, lipids and other intracellular molecules leading to apoptosis or necrosis of tumor cells (Kubiak et al., 2016; Duse et al., 2018). Besides direct cytotoxicity, PDT can also target tumor vasculature, resulting in blood flow stasis and vessel constriction or collapse. Subsequent tissue hypoxia and lack of nutrients lead to tumor cell death (Wang et al., 2012). Additionally, some photosensitizers induce tissue devastation accompanied by acute inflammatory processes, followed by a substantial anti-tumor immune response (Yang et al., 2016). Hypericin, a plant-derived pigment, triggers the exposure of damage-associated molecular patterns (DAMPs) on cancer cells, inducing recognition by the innate immune system and phagocytosis of damaged cancer cells (Garg et al., 2012). Furthermore, hypericin preferentially localizes in the endoplasmic reticulum (ER) which is known to play a key role in immunogenic cell death (ICD) (Garg & Agostinis, 2014). Overall, hypericin is an attractive photosensitizer for daily clinical practice due to its activity in all three PDT cell death pathways, minimal dark toxicity, rapid clearance from normal healthy tissue and selective accumulation in tumor cells (Davids, 2012). Moreover, it can also act as a fluorescence marker for diagnostic imaging (Gattuso et al., 2017). Nevertheless, hypericin exhibits a very hydrophobic character, making it impossible to apply therapeutic doses (Abrahamse & Hamblin, 2016). The photosensitizer tends to aggregate in aqueous milieu leading to low bioavailability and the loss of photodynamic activity (Kubin et al., 2005). To overcome these limitations, hypericin can be encapsulated into liposomes. Liposomes are biocompatible, able to carry large amounts of drug and show a wide range of physicochemical and biophysical characteristics that can be modified to define their biological fate (Sercombe et al., 2015). As previously reported, stable incorporation of hypericin into liposomes cannot be achieved easily, since the PS has a high affinity to albumin and lipoproteins (Derycke & Witte, 2002). To avoid premature photosensitizer release into the bloodstream and assure transport to the tumor site, liposomes with increased membrane rigidity are required. By incorporating tetraether lipids (TEL) derived from the plasma membrane of thermoacidophilic archaebacterium *Sulfolobus acidocaldarius* into the vesicles this characteristic can be provided (Mahmoud et al., 2017). The bipolar TEL molecules consist of two biphytanyl chains attached to hydrophilic headgroups by ether linkages at both ends. In the present study a mixture of caldarchaeol (glycerol dialkyl glycerol tetraether, GDGT) which is linked to two glycerols at both ends of the hydrophobic core and calditoglycerocaldarchaeol (glycerol-dialkyl-nonitol-tetraether, GDNT) which is attached to a glycerol backbone at one end of the hydrophobic moiety and a calditol group at the other end were used. Compared to monopolar diester lipids TEL molecules are stretched and span the liposomal membrane. This monolayer organization in addition to the branching methyl groups in the saturated chains results in more rigid membranes and less permeability (Jacquemet et al., 2009).

**Scheme 1.**
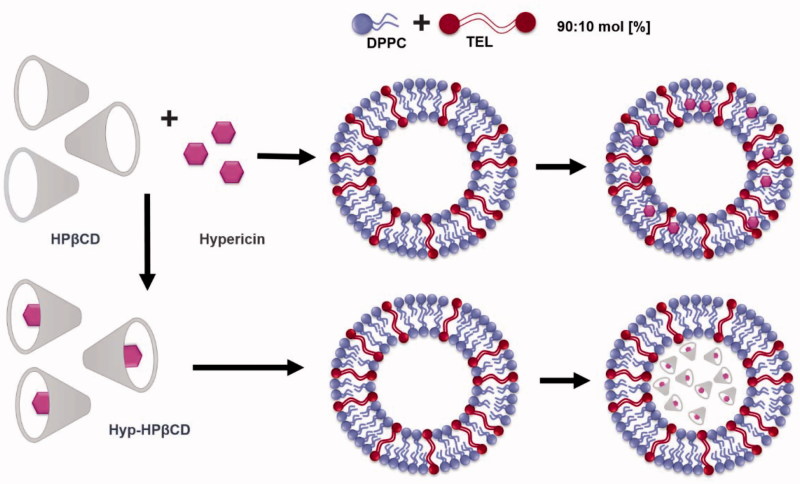
Preparation of an inclusion complex (Hyp-HPβCD) consisting of hypericin and hydroxypropyl-β-cyclodextrin (HPβCD). Empty liposomes are loaded with either hypericin which associates with the hydrophobic tails in the lipid membrane or with Hyp-HPβCD incorporated in the aqueous compartment of liposomes.

In this study, conventional liposomes composed of 1,2-distearoyl-sn-glycero-3-phosphatidyl-choline (DSPC) and tetraether liposomes composed of 1,2-dipalmitoyl-sn-glycero-3-phosphatidyl-choline (DPPC)/TEL were loaded either with hypericin through a thin film hydration method or with hypericin-hydroxypropyl-β-cyclodextrin inclusion complex (Hyp-HPβCD) via dehydration-rehydration vesicle method (Scheme 1). The liposomes were characterized and compared according to size, zeta potential, morphology, stability, and encapsulation efficiency. Furthermore, hemocompatibility was tested by hemolysis and coagulation time test. The aim of this study was to compare the differences between conventional and TEL liposomes as carriers for hypericin and to highlight the particular advantages of the two different encapsulation strategies. The impact of lipid composition and intraliposomal hypericin localization on the direct phototoxicity on human ovarian carcinoma cells was investigated. Furthermore, after intravenous injection, the antiangiogenic effect of hypericin liposomes was evaluated in the chorioallantoic membrane (CAM) model.

## Materials and methods

### Materials

Hypericin was purchased from Thermo Fisher Scientific (Karlsruhe, Germany). 1,2-distearoyl-*sn*-glycero-3- phosphatidyl-choline (DSPC) and 1,2-dipalmitoyl-sn-glycero-3-phosphatidyl-choline (DPPC), were a gift from Lipoid GmbH (Ludwigshafen, Germany). A polar lipid fraction containing tetraether lipids caldarchaeol (GDGT ∼10% of the extract) and calditoglycerocaldarchaeol (GDNT ∼90% of the extract) was extracted from the biomass of *S. acidocaldarius* (SIT Rosenhof GmbH, Heiligenstadt, Germany) as previously mentioned (Engelhardt et al., 2017). (2-hydroxypropyl)-β-cyclodextrin (HPβCD) and all other chemicals and solvents used were of analytical grade and were purchased from Sigma-Aldrich (Taufkirchen, Germany).

### Preparation of hypericin liposomes

#### Preparation of empty and hypericin liposomes by thin film hydration method

Empty liposomes were prepared according to the thin film hydration method, with two different lipid compositions: DPPC/TEL in molar ratio of 90/10 and pure DSPC. The lipids dissolved in chloroform:methanol (2:1 (v/v)) were mixed in a round-bottom flask in total amount of 10 µmol/ml of lipid and dried by rotary evaporation (Heidolph Laborota 4000 efficient, Heidolph Instruments, Schwabach, Germany). The rehydration of the obtained lipid films was performed with PBS buffer (pH 7.4) followed by sonication for 10 min. To obtain hypericin liposomes an appropriate amount of hypericin stock solution in methanol was added to the lipid mixture before evaporation.

#### Preparation of Hyp-HPβCD-complex

The formation of Hyp-HPβCD inclusion complex was performed according to a method previously described, with slight modifications (McCormack & Gregoriadis, 1994). Briefly, hypericin dissolved in methanol was dried in a round bottom flask by rotary evaporation until the formation of a thin hypericin film. HPβCD-solution of about 5% was added to the film followed by 30 min sonication. After stirring for a definite time, the Hyp-HPβCD complex solution was purified by filtration through a 0.22 µm nylon filter. To calculate the amount of hypericin bound to HPβCD, the solution was diluted in methanol and the fluorescence was measured.

#### Preparation of DRV’s

For encapsulation of Hyp-HPβCD-complex into liposomes, the dehydration-rehydration vesicle (DRV) method was used (Antimisiaris, 2010). Briefly, 1 ml of empty liposomes and a volume of Hyp-HPβCD-complex containing 200 µg of hypericin were added in a round bottom flask. The mixture was freeze-dried and rehydrated in 200 µl of MilliQ water. After 30 min of incubation in a water bath (60 °C), 800 µl of PBS buffer (pH 7.4) was added. The non-entrapped complex was separated by size exclusion chromatography using Sephadex G-25 (PD-10 Columns, GE Healthcare, Germany).

### Characterization of hypericin liposomes

#### Photon correlation spectroscopy (PCS) and laser Doppler velocimetry (lDv)

The hydrodynamic diameter and zeta potential of the liposomes were measured by PCS and lDv respectively using Zetasizer Nano ZS (Malvern Instruments, Herrenberg, Germany) equipped with 10 mW HeNe laser at a wavelength of 633 nm at 25 °C. Laser attenuation and measurement position were adjusted automatically by the instrument. The zeta potential was measured through electrophoretic mobility with laser Doppler velocimetry (lDv). The average values of the size intensity peak and zeta potential were calculated with data of three independent experiments ± standard deviation. Each sample was measured three times with at least 10 sub runs.

#### AFM measurements

AFM was performed using a NanoWizard® 3 atomic force microscope (JPK Instruments, Berlin, Germany). Silicon cantilevers (HQ:NSC14/AL_BS, MikroMasch Europe, Wetzlar, Germany) were used to measure the samples. Measurements were performed in intermittent contact mode to avoid damage to the liposomes (Sitterberg et al., 2010). Images were obtained by displaying the amplitude signal of the cantilever in the trace direction and the measured height mode in retrace direction for which a cross-sectional profile along the identified lines is presented.

#### Entrapment efficiency

Free hypericin or Hyp-HPβCD-complex was separated from the liposomes by size exclusion chromatography using Sephadex G-25. Before adding Hyp-HPβCD liposomes, the column was saturated with empty liposomes of the same lipid formulation. The liposomes obtained after SEC were diluted in methanol and the fluorescence intensity of the solution was measured (FLUOstar, BMG, Germany), using an excitation wavelength of 540 nm and an emission wavelength of 590 nm. The amount of hypericin was calculated according to Equation (1) using a calibration curve of hypericin in methanol.

(1) $$\text{EE}\;\left(\%\right)=\frac{\text{hypericin}\;\text{amount}\;\text{encapsulated}}{\text{hypericin}\;\text{amount}\;\text{added}}\times 100$$

#### Stability in IMDM

In order to assess the stability of liposomes in cell culture medium, 900 µl IMDM supplemented with 10% FBS was mixed with 100 µl of hypericin liposomes. The samples were incubated at 37 °C. After definite time intervals samples were diluted 1:10 with MilliQ water for measurement of hydrodynamic diameter, PDI and zeta potential. Results were calculated with data of three independent experiments ± standard deviation.

### Cell culture experiments

#### Cell lines and cell culture

Wild-type human ovarian adenocarcinoma cells (SK-OV-3) were purchased from ATCC (Virginia, USA) and cultured in IMDM medium (Capricorn Scientific, Ebsdorfergrund, Germany) supplemented with 10% fetal bovine serum (FBS; Sigma Aldrich). Cells were grown as monolayers at 37 °C and 7% CO_2_ under humid conditions and sub-cultured upon reaching 80% confluency.

#### Irradiation experiments

The cells were seeded into 96 well plates (NUNC, Thermo Scientific, Germany) at a cell density of 10 000 cells/well. After 24 h, the cells were incubated with different concentrations of hypericin liposomes for 1, 2 and 4 h. After incubation, the liposomes were replaced by fresh IMDM and irradiated with a LED-device at 587 nm (Lumundus, Eisenach, Germany). Subsequently, the cells were incubated 24 h. Cytotoxicity was determined by MTT assay. The medium was aspirated and 3-(4,5-Dimethylthiazol-2-yl)-2,5-Diphenyltetrazolium bromide (MTT) was added to each well. Following, the cells were incubated for 4 h in the dark. Viable cells convert the water-soluble MTT to a water-insoluble purple formazan, which can be solubilized in DMSO and quantified by measuring the absorption at 570 nm using a plate reader (FLUOstar, BMG, Germany). Blank values represent 100% viability. The average of three wells per concentration was taken. Empty liposomes with and without irradiation as well as hypericin liposomes without irradiation were used as control samples.

#### Pathway studies

SK-OV-3 were seeded into 96 well plates at a cell density of 10 000 cells/well and maintained at 37 °C for 24 h. After a pre-incubation period of 60 min with Filipin III (10 µg/ml) or chlorpromazine (10 µg/ml), hypericin liposomes with a total hypericin concentration of 250 nM were added. After 3 h incubation, the liposomes were replaced by fresh medium and the cells were irradiated with 8.3 J/cm^2^. Subsequently, the cells were incubated for 24 h and the cell viability was determined by MTT assay as described above. A non-irradiated plate served as control.

### Hemocompatibility studies

#### Activated partial thromboplastin time (aPTT)

The aPTT test was performed to investigate the influence of the different liposomal formulations on blood coagulation. Fresh blood was drawn into citrate tubes followed by centrifugation at 1000 g for 10 min to separate the plasma fraction. The aPTT test was performed in a Coatron M1 coagulation analyzer (Teco, Neufahrn, Germany) using the TEClot aPTT-S Kit as per the manufacturer’s protocol with slight modifications (Pinnapireddy et al., 2017). Briefly, 25 μL of plasma was mixed with 25 μL of sample. 25 μL of aPTT reagent was added to the sample to activate coagulation factors followed by the addition of pre-warmed calcium chloride. Coagulation was confirmed spectrophotometrically and the time was recorded in seconds.

#### *Ex vivo* hemolysis assay

To determine the hemolytic potential of the liposomes, human erythrocytes were isolated from fresh blood as described previously (Evans et al., 2013). First, fresh blood was drawn into tubes containing EDTA followed by centrifugation of the whole blood. The obtained red blood cell pellet was washed thrice with 0.9% NaCl (pH 7.4) and diluted to 1:50 with 0.9% NaCl. The erythrocytes were incubated together with the formulations for 1 h at 37 °C in v-bottom microtitre plates in an orbital shaker (KS4000 IC, IKA Werke, Staufen, Germany). The plates were then centrifuged and the absorbance of the collected supernatant was determined at 540 nm in a FluoStar Optima plate reader. PBS buffer (pH7.4) and 1% Triton X-100^®^ were used as controls and the absorbance values of Triton X-100^®^ were considered as 100% hemolysis.

### Confocal laser scanning microscopy

CLSM was used to visualize the uptake of hypericin into SK-OV-3 cells. SK-OV-3 cells were incubated overnight in 12 well plates (90 000 cells/well) with coverslips (15 mm diameter). On the following day, the cells were incubated for 4 h with hypericin liposomes at a final concentration of 1 µM. The cells were subsequently washed with PBS containing Ca^2^/Mg^2+^ (pH 7.4) before being fixed with 4% paraformaldehyde for 20 min. The cells were washed again with PBS containing Ca^2^/Mg^2+^ (pH 7.4) and counterstained with DAPI (0.1 µg/ml). After another washing step, the coverslips were transferred onto glass slides and mounted using FluorSave™ (Calbiochem Corp, La Jolla, CA). The samples were examined with CLSM (LSM 700; Carl Zeiss, Jena, Germany) using 405 and 555 nm laser lines (for DAPI and hypericin respectively) and the images were acquired using the ZEN software (Carl Zeiss).

### Chorioallantoic membrane (CAM) experiments

Fertilized chicken eggs were purchased from Brormann GmbH (Rheda-Wiedenbrück, Germany) and were incubated in a hatching incubator equipped with an automatic rotator (Dipl. Ing. W. Ehret GmbH, Emmendingen, Germany) at 37 °C and a relative humidity of 60%. The CAM angiogenesis model was prepared as previously described with slight modifications (Özcetin et al., 2013). On egg development day 4 (EDD 4) the eggs were punched on the blunt side with a pneumatic EggPunch (Schuett-Biotec, Germany) and the eggshell was removed along the fracture line to expose part of the CAM in a round window of 30 mm diameter. The hole was covered with small sterile petri-dish to avoid contamination and the eggs were placed in a stationary incubator until EDD 12. After intravenous injection of 100 µl liposomes containing 100 µM hypericin, a small polypropylene ring was placed on the CAM surface in order to specify the treated spot. The liposomes were incubated for 7 min and the specified area was irradiated with the Weber needle Endolaser (Weber medical, GmbH, Lauenförde, Germany) at 589 nm and a radiation fluence of 11.4 J/cm^2^. Images monitoring the photodynamic effect on the microvasculature were taken before irradiation, directly and 60 min after irradiation with a Stemi 2000-C stereomicroscope (Carl Zeiss) equipped with a Moticam 5 CMOS camera (Motic Deutschland, Wetzlar, Germany). The experiment was performed three times for each liposomal formulation and eggs treated with 0.9% NaCl solution served as a negative control. The treatment effect was categorized into different levels according to criteria previously described (Pegaz et al., 2006). The different levels describe the degree of vascular damage.

### Statistical analysis

All experiments were performed in triplicates and the values are presented as mean ± standard deviation unless otherwise stated. Two-tailed Student’s *t*-test was performed to identify statistical significance differences. Probability values of *p* < .05 were considered significant. Statistical differences are denoted as ‘∗’ *p* < .05, ‘∗∗’ *p* < .01, and ‘∗∗∗’ *p* < .001.

## Results and discussion

In this study, a parenteral formulation consisting of liposomes encapsulating either hypericin or Hyp-HPβCD inclusion complex was prepared in order to create an effective photodynamic treatment of tumors. Firstly, a lipid film containing hypericin was rehydrated and the photosensitizer was associated with the hydrophobic tails in the lipid membrane. Secondly, a mixture of an inclusion complex of hypericin with 2-hydroxypropyl-β-cyclodextrin (HPβCD) and empty liposomes was lyophilized. During this process, the empty multilamellar vesicles (MLV) were disrupted. Consequently, a specific rehydration protocol led to high entrapment of Hyp-HPβCD into the aqueous compartment of the fused liposomes. All formulations were characterized and compared regarding their size, zeta potential, morphology, encapsulation efficiency, stability, and cellular uptake. The focus of this study lies on the photodynamic efficiency of the formulations on SK-OV-3 cells and the photodestructive effect on the microvasculature of the CAM as a function of lipid composition and intraliposomal photosensitizer localization.

### Characterization of hypericin liposomes

#### Physicochemical and morphological characterization of liposomes

**Figure 1.**
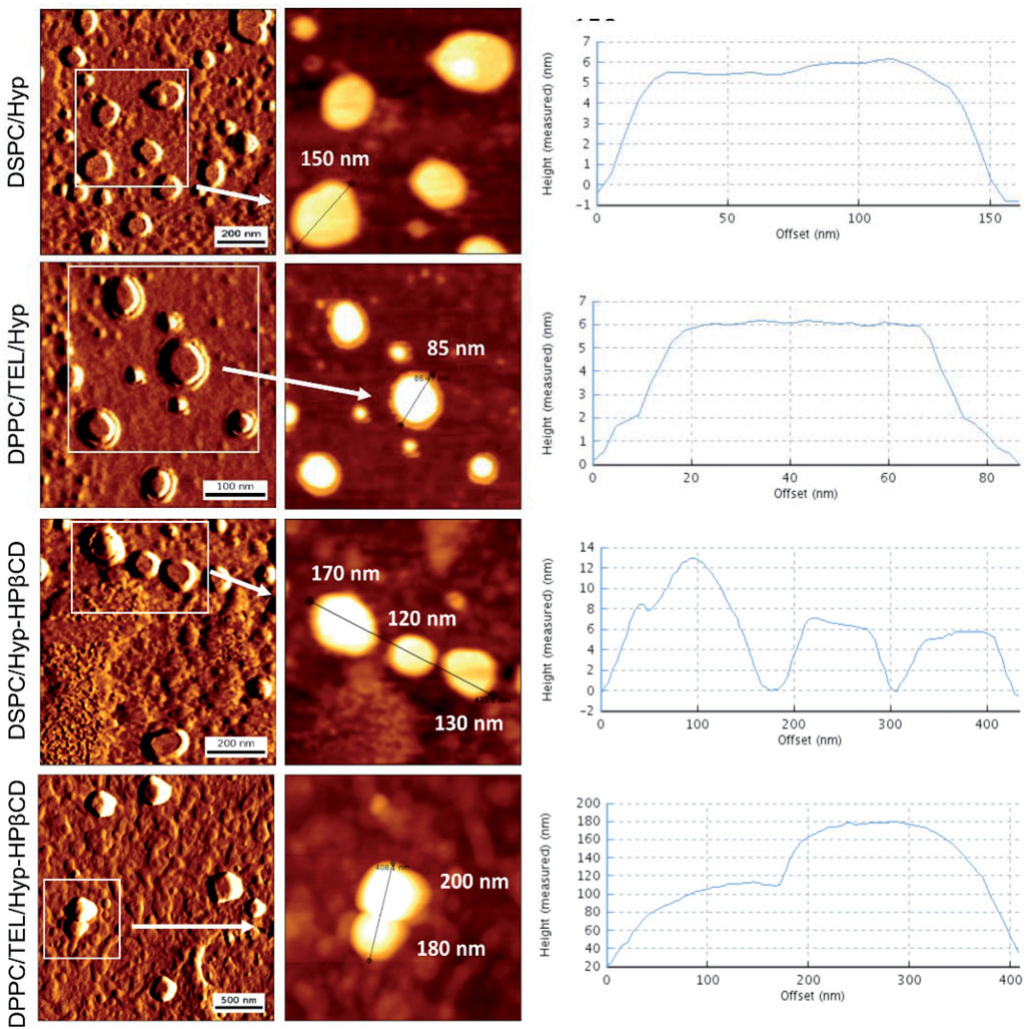
Visualization of liposomes by AFM. Images shown on the left side are displayed in amplitude mode while the middle row shows images in measured height mode for which a cross-sectional profile along the identified lines is presented.

AFM measurements indicated spherically shaped liposomes (Figure 1). The liposomal diameters resulting from the evaluation of images in the measured height mode are in accordance with the DLS measurements shown in Table 1. The prepared liposomes exhibited hydrodynamic diameters between 127 ± 14 and 212 ± 13 nm. DPPC/TEL liposomes encapsulating only hypericin showed a smaller hydrodynamic diameter than DPPC/TEL vesicles containing the inclusion complex. Furthermore, the zeta potential was much lower for both lipid compositions when Hyp-HPβCD was incorporated. We assume that not all of the Hyp-HPβCD complex can be removed by SEC. Consequently, the negatively charged inclusion complex also adheres to a small amount on the liposomal surface. The polydispersity index (PDI) between 0.26 and 029 refers to a polydisperse size distribution of the prepared liposomes.

**Table 1.** Hydrodynamic diameter, polydispersity index (PDI), zeta potential and entrapment efficiency (EE) [%] of hypericin liposomes. Hydrodynamic diameter is expressed as a measure of particle size distribution by intensity, *n* = 3.

Lipid composition	Diameter ± SD [nm]	PDI	Zeta potential ± SD [mV]	Theoretical load [µg/ml]	Practical load [µg/ml]	EE [%]
DSPC/Hyp	150 ± 11	0.26	–1 ± 1	150	50.5 ± 6.2	33.6 ± 4.1
DSPC/Hyp-HPβCD	169 ± 10	0.26	–22 ± 1	200	129.6 ± 7.8	64.8 ± 3.9
DPPC/TEL/Hyp	127 ± 14	0.24	–2 **±** 1	150	123.8 ± 4.2	82.5 ± 2.8
DPPC/TEL/Hyp-HPβCD	212 ± 13	0.29	–56 ± 1	200	163.2 ± 6.4	81.6 ± 3.2

#### Entrapment efficiency

The drug-in-cyclodextrin liposomes achieved an entrapment of 64.8% for DSPC and 81.6% for DPPC/TEL liposomes. While the carriers incorporating hypericin showed an encapsulation of 33.6% for DSPC and 82.5% for DPPC/TEL liposomes (Table 1). These findings indicate that the encapsulation of hypericin as inclusion complex instead of free hypericin resulted in higher entrapment efficiencies for DSPC carriers. It has been shown that hypericin tends to form aggregates in giant unilamellar vesicles (GUV) (Joniova et al., 2017). Conclusively, it can be assumed that the formation of hypericin clusters which are removed by SEC lead to a reduced amount of hypericin bound to the DSPC membrane. Additionally, both DPPC/TEL liposomes were encapsulating higher amounts of hypericin when compared to their DSPC counterparts. This can be explained by the high stability of the liposomal membrane upon addition of TEL lipids leading to a decreased loss of the encapsulated material (Uhl et al., 2017). Furthermore, entrapment efficiencies of DSPC/Hyp liposomes are comparable to literature data, while for TEL vesicles, it was possible to achieve a higher entrapment efficiency of hypericin than previously reported (Galanou et al., 2008). Thus the unique characteristics of the TEL liposome membrane seem to enhance the entrapment efficiency for both encapsulation strategies.

#### Stability of hypericin liposomes

To ascertain the stability of the liposomes during cell culture experiments, PCS and lDv measurements of liposomes incubated in IMDM supplemented with 10% FBS at 37 °C were carried out. Table 2 shows the results directly, 1 and 24 h after incubation with the cell culture medium. The PDI’s indicate that all formulations are stable in IMDM. However, a slight increase of the PDI indicates that the size distribution of vesicles is relatively broad. Additionally, the hydrodynamic diameters increased for all formulations. These phenomena might be explained by the formation of a protein corona surrounding the liposomes. It was demonstrated that liposomes of varying compositions increased between 12 and 138 nm in hydrodynamic diameter and in PDI after incubation in human plasma (Bigdeli et al., 2016). Furthermore, the negative zeta potential of the liposomes encapsulating Hyp-HPβCD complex increased compared to the zeta potential measured in the buffer. This ‘normalization’ of the zeta potential is another indication for the presence of a protein corona. Thereby liposomes with charged surfaces adsorb more proteins than neutral ones, but the surface charge is not the only criteria for corona formation. Another important factor is the interaction between the proteins and lipid functional groups on the surface (Caracciolo, 2015). Regarding the changes of zeta potential from storage in the buffer to incubation in cell culture medium, DPPC/TEL liposomes tend to adsorb more proteins than their counterparts consisting of DSPC. The adsorption of proteins can, among other things, alter the circulation time of liposomes. Complement factors, fibrinogen, or IgG are so-called opsonins which enhance phagocytosis, while dysopsonins like albumin or apolipoproteins prolong the circulation time (van Nguyen & Lee, 2017). Since the characteristics of the corona are highly dependent on lipid composition, every liposome forms an individual corona (Caracciolo et al., 2014). This should be considered for further investigations.

**Table 2.** Stability of hypericin liposomes incubated in IMDM (IMDM:liposomes/10:1 [v/v]). Hydrodynamic diameter is expressed as a measure of particle size distribution by intensity, *n* = 3.

Formulation	Time [h]	Diameter ± SD [nm]	PDI ± SD	Zeta potential ± SD [mV]
DSPC/Hyp	0	297 **±**26	0.34 ± 0.03	–4.4 ± 1
DSPC/Hyp-HPβCD	1	346 ± 10	0.46 ± 0.04	–4.3 ± 1
	24	239 ± 21	0.41 ± 0.04	–3.6 ± 1
	0	409 ± 13	0.42 ± 0.02	–6.5 ± 1
	1	520 ± 15	0.48 ± 0.03	–7.2 ± 1
	24	473** **±26	0.43** **±0.02	–6.8 ± 1
DPPC/TEL/Hyp	0	235 ± 14	0.35 ± 0.09	–8.5 ± 1
	1	237** **±17	0.37 ± 0.08	–9.2 ± 1
	24	366 ± 28	0.53 ± 0.06	–10.1 ± 1
DPPC/TEL/Hyp-HPβCD	0	232 ± 31	0.48 ± 0.03	−13.8 ± 1
	1	204 ± 20	0.54 ± 0.05	–16.3 ± 1
	24	268 ± 36	0.45 ± 0.04	–15.6 ± 1

### CLSM measurements

**Figure 2.**
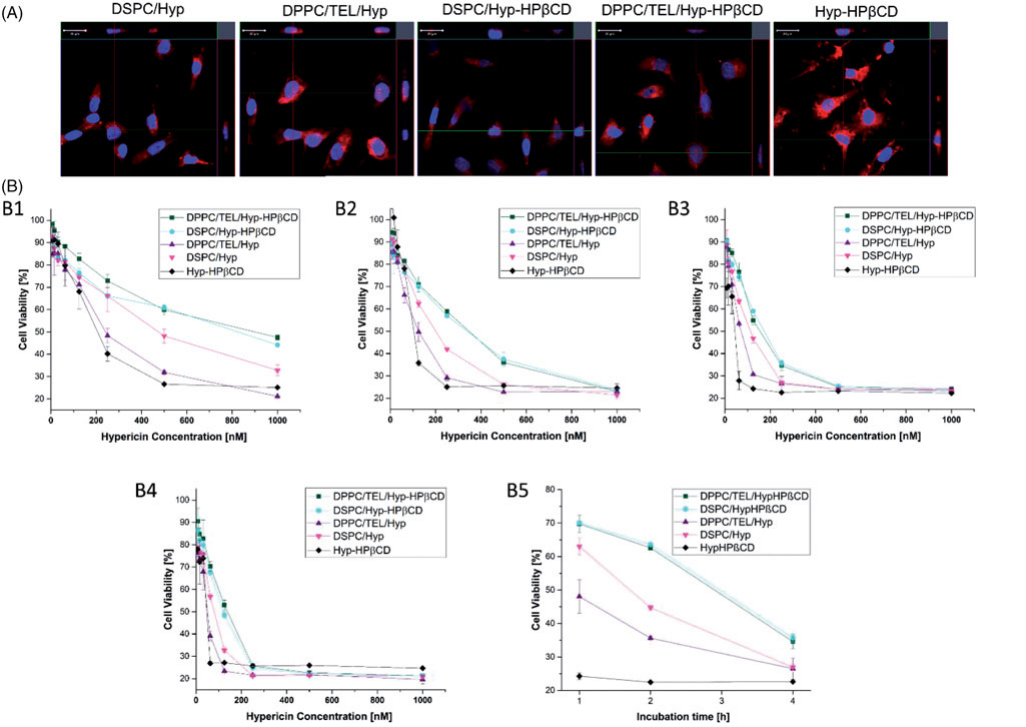
(A) CLSM images of SK-OV-3 cells incubated with Hyp-HPβCD or liposomes consisting of DSPC or DPPC/TEL (90/10 mol:mol) encapsulating either hypericin or Hyp-HPβCD inclusion complex in a final concentration of 1 µM (B) SK-OV-3 cells incubated with different concentrations of free Hyp-HPβCD inclusion complex or liposomes. Incubation time was 4 h and the cells were irradiated with different light fluences of 2.1 J/cm^2^ (B1), 4.1 J/cm^2^ (B2), 8.3 J/cm^2^ (B3) and 12.4 J/cm^2^ (B4). B5 shows the influence of the incubation time (1, 2 and 4 h) on photocytotoxicity of hypericin nanoformulations with a final concentration of 250 nM hypericin and a radiation fluence of 8.3 J/cm^2^.

For the visualization of hypericin uptake into SK-OV-3 cells confocal microscopy was utilized (Figure 2(A)). After 4 h incubation with 1 µM of the different formulations, a considerable hypericin accumulation in the cells could be observed. Yet there was no difference between the individual liposome formulations. Only for free Hyp-HPβCD a more intense fluorescence was detected. Even if the *in vitro* uptake of free Hyp-HPβCD seems to be higher than those of liposomes, it is less targeted. Furthermore, the *in vivo* fate of 90% of the intravenously applied hydroxyl-propyl-ß-cyclodextrins is the clearance into the urine after 4 h and complexed lipophilic compounds would rapidly bind to proteins and lipoproteins of the serum (Pitha et al., 1994). Thus the encapsulation into liposomes is necessary for a controlled tumor targeting and *in vivo* behavior.

### Phototoxic effect on ovarian carcinoma cells

The direct photo-cytotoxic effect of the different liposomal formulations of hypericin and Hyp-HPβCD was evaluated on SK-OV-3 a human ovarian carcinoma cell line. Figure 2(B1–B4) shows a dose-dependent phototoxic effect of all liposomal formulations and free Hyp-HPβCD at an irradiation fluence of 2.1, 4.1, 8.3, and 12.4 J/cm^2^ after 4 h incubation. Since the localization of the photosensitizer in the cell is crucial for successful photodynamic treatment, it can be assumed that also the uptake of hypericin in SK-OV-3 is dose dependent (Castano et al., 2004). This observation and the dosage range are in accordance with literature data (Mühleisen et al., 2017). For all formulations, a dark control was made to ensure that the detected toxicity was due to light absorbance. The highest photo-toxicity was found for DPPC/TEL/Hyp liposomes and free Hyp-HPβCD at an irradiation fluence of 12.4 J/cm^2^ leading to IC_50_ values of 48 and 42 nM respectively (Supplementary material). Since DSPC/Hyp liposomes showed an IC_50_ value which was twice that of DPPC/TEL/Hyp, we assume that TEL-liposomes are delivering more hypericin to the target site than their counterparts consisting of DSPC. The photocytotoxicity of DPPC/TEL/Hyp-HPβCD and DSPC/Hyp-HPβCD vesicles was lower compared to liposomes incorporating hypericin in the membrane with an IC_50_ value of 136 and 120 nM for 12.4 J/cm^2^ respectively. However, the cell viability after liposomal PDT treatment approached a similar level for all liposomal formulations after increasing the radiation fluence stepwise from 2.1 to 12.4 J/cm^2^ (Figure 2(B1–B4)). These findings may indicate that hypericin is better protected from light by encapsulation as inclusion complex in liposomes. This supports the previous suggestion to utilize the drug-in-CD-in-liposome system for protection of photosensitive drugs (Ioele et al., 2017). Figure 2(B5) shows that the photo-destruction of SK-OV-3 cells with free HPβCD was not depending on incubation time. In contrary, the duration of liposome exposure was essential for the photodynamic action of the same. This effect was the most obvious for liposomes encapsulating the inclusion complex. This observation is again pointing towards a more stable encapsulation in the drug-in-CD-in-liposome system.

### Pathway studies

**Figure 3.**
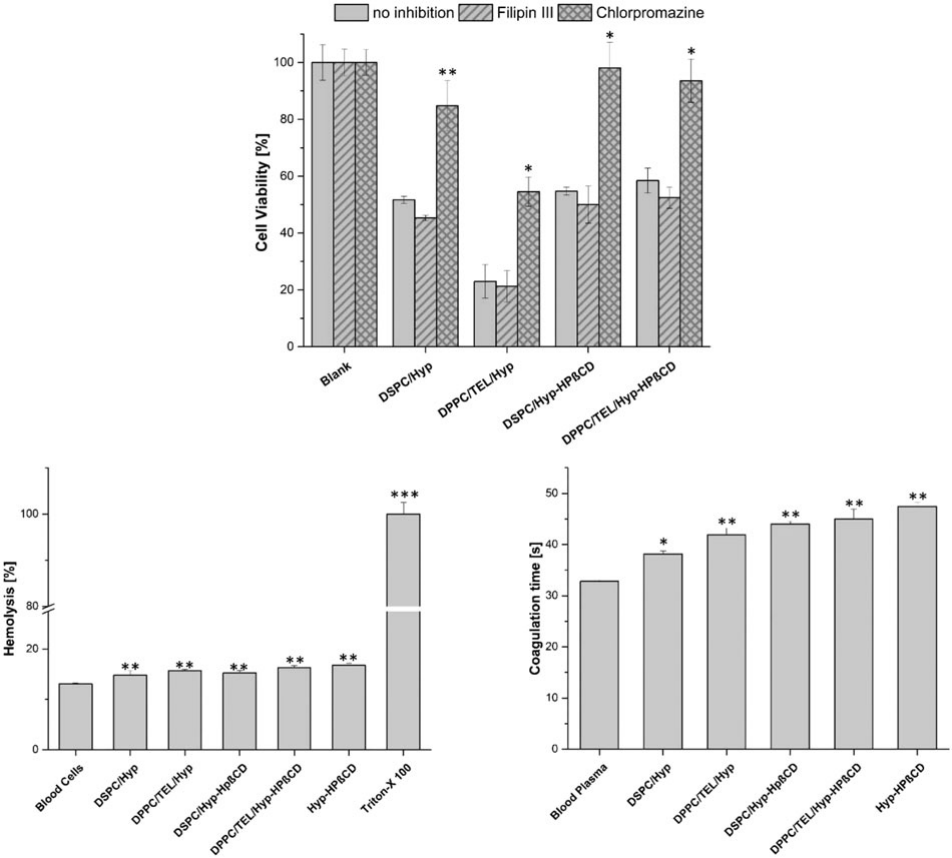
(A) Cell viability of SK-OV-3 cells after incubation with medium (Blank) or different liposomal formulations (250 nM hypericin) for 3 h and irradiation at 8.3 J/cm^2^. The endocytotic pathway was unhampered (no inhibition) or inhibited by Filipin III or Chlorpromazine. For statistical analysis, the results were compared against the ‘no inhibition’ value. (B) Hemolysis assay of the different liposomal formulations. Triton™ X-100 was used as positive control. (C) aPTT test of the different liposomal formulations. Blood plasma was used as control. All samples were measured in triplicates and results are expressed as the mean ± SD (*n* = 3) For statistical analysis, the results were compared against the results of not inhibited cells, blood cells, and fresh blood plasma respectively.

To examine the mechanism of liposomal internalization into SK-OV-3 cells, endocytosis pathways were inhibited. Chlorpromazine and Filipin III were utilized to suppress the clathrin-mediated and the caveolae-mediated endocytosis respectively. Dark-control cells which were unirradiated and incubated with inhibitors and liposomal formulations, showed no decrease in cell viability. Figure 3(A) demonstrates that inhibitors alone had no effect on cell viability despite irradiation. Cells which were incubated with liposomal formulations without inhibitor could take up the liposomes and showed a corresponding decrease in cell viability. Additional pre-incubation with Filipin III showed no inhibition of liposomal uptake, while pre-incubation with chlorpromazine inhibited the uptake of all liposomal formulations. The impact of chlorpromazine was particularly strong for liposomes encapsulating the inclusion complex, indicating that the uptake occurs mainly by clathrin-mediated endocytosis. These findings are supported by other workgroups stating that up to a size of ∼200 nm the preferential uptake pathway is the formation of clathrin-coated pits (Rejman et al., 2004). For DSPC/Hyp and DPPC/TEL/Hyp liposomes other possibilities of liposome-cell interaction can be considered, such as the adsorption of liposomes on the cell surface and the release of hypericin in the extracellular compartment (Lasic, 1993). So it can be also assumed that hypericin released from the liposomes could target the cell membranes through diffusion or by LDL-pathway (Ho et al., 2009).

### Hemocompatibility

Since the ideal liposomes are carrier systems with long circulation times in the bloodstream in order to deliver their cargo to tumor cells, hemocompatibility studies are of great importance. Furthermore, they can contribute to a better understanding of the relations between *in vitro* and *in vivo* results and can lead to a safe *in vivo* concentration. Therefore, we investigated the impact of all formulations on blood by carrying out hemolysis and aPTT tests. Figure 3(B) shows the results of the hemolysis assay which determines the release of hemoglobin from erythrocytes upon exposure to formulations. The hemolytic toxicity of the tested liposomes was negligible. The aPTT measurements provide insights into the effect of the hypericin formulations on blood coagulation (Figure 3(C)). The coagulation time for normal plasma was 32.8 s, which was increased by free Hyp-HPβCD inclusion complex by only 14.7 s. The liposomal formulations showed an increase in coagulation time between 5 and 12 s. An aPTT value between 30 and 40 s is acceptable while values over 70 s imply continuous bleeding (Pinnapireddy et al., 2017).

### Phototoxic effect on microvasculature

**Figure 4.**
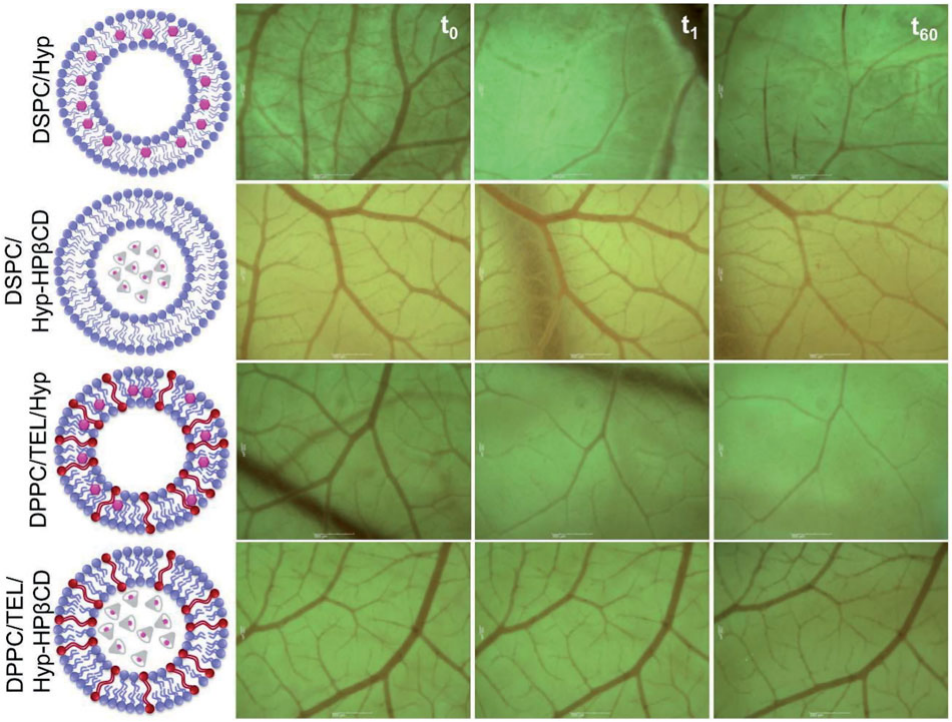
Tabular display of the devastation of CAM microvasculature after intravenous injection of 100 µl liposomes consisting of DSPC or DPPC/TEL (90/10 mol:mol) encapsulating either hypericin or Hyp-HPβCD inclusion complex in a total concentration of 100 µM before irradiation (*t*0), shortly after irradiation (*t*1) and 60 min after irradiation (*t*60) with a low level laser of 589 nm at an irradiation fluence of 11.4 J/cm^2^.

The main source of nutrients and oxygen for solid tumors is their own vascular system, which they establish upon reaching a definite size (Abels, 2004). Damage to endothelial cells during PDT treatment induces thrombus formation within the vessel lumen, which triggers a physiological cascade of responses. Thereby the vascular permeability increases and the vessels constrict or collapse, leading to blood flow stasis (Fingar, 1996). Thus targeting the vascular system of tumors is the second line of action of PDT. In order to investigate vascular events during photodynamic treatment, CAM model was used. Since the CAM is a transparent membrane, it is possible to investigate individual blood vessels and monitor the uptake of formulations on the stasis or blood flow in real time. At first, it was ensured that light alone or light in combination with the injection of empty liposomes or PBS buffer had no effect on the integrity of the microvasculature (data not shown). Figure 4 shows the impact on microvasculature of the different liposomal formulations with a hypericin-light interval of 7 min. DSPC/Hyp liposomes exhibited a substantial photo-destruction of the microvasculature resulting in treatment level 3 which indicates a total closure of small vessels (ø < 30 µm) and partial closure of bigger vessels. However, DPPC/TEL/Hyp liposomes were inducing a level 4 reaction with a total collapse of bigger vessels (ø < 70 µm). Whereas for DSPC/Hyp-HPβCD liposomes only a slight decline in blood flow rate and a closure of small capillaries (ø < 10 µm) could be observed (Figure 4). No difference in the perfusion of the vasculature or vesicle integrity could be found for DPPC/TEL/Hyp-HPβCD liposomes. The optimal range of microvasculature damage is between level 3 and 4, since a total shutdown of tumor vasculature would affect the oxygen-dependent photodynamic process (Gihan Neues Paper). These results indicate that DPPC/TEL/Hyp liposomes lead to sufficient and rapid hypericin accumulation in endothelial cells and in the bloodstream. Perhaps one reason might be the partial release of hypericin from liposomes due to its high affinity to plasma proteins, which also in case of albumin and LDL serve as carriers for the photosensitizer. Therefore, DPPC/TEL/Hyp liposomes not only show good characteristics for cellular tumor targeting but also for vascular targeting. Drug-in-cyclodextrin liposomes in contrary encapsulate the photosensitizer in a stable manner and show only moderate anti-vascular effect under the current conditions. Moreover, TEL lipids are able to enhance this characteristic and shield the photosensitizer completely from light. This may lead to longer circulation times. The formation of a liposome corona can contribute to this event. Hence findings in the stability studies need to be further investigated, in order to determine the type of the resulted corona. Since the photochemical cytotoxic effect of all formulations is in a therapeutic range these results indicate that the drug-in-cyclodextrin carriers might be suitable for delivering the cargo to the tumor site after intravenous application.

## Conclusion

In this study, liposomal formulations of hypericin solved the issue of poor solubility of the same. Liposomes make hypericin available as aqueous preparation in therapeutic doses suitable for intravenous application. This was confirmed by the hemocompatibility studies. Furthermore, incubation in cell culture media proved the stability of the liposomes. The prepared formulations showed no dark toxicity on SK-OV-3 cells, but a considerable photodestructive effect after irradiation. Since hypericin tends to accumulate preferentially in tumor tissue and is only activated by light, PDT offers a tumor-targeted therapy. This effect can be enhanced by the application of liposomes, since they can prolong the circulation time of hypericin and Hyp-HPβCD. Thereby the two different liposomal types determined by the particular encapsulation strategy for hypericin point toward different capabilities. On the contrary, conventional hypericin liposomes seem to be a good choice for vascular targeting of the tumor as demonstrated in the CAM experiments. While on the other hand liposomes encapsulating the Hyp-HPβCD inclusion complex could be suitable for delivering hypericin directly to the tumor cells by very stable incorporation of the cargo. In doing so liposomes consisting of TEL lipids seem to be superior to liposomes consisting of conventional lipids like DSPC. Our prime research aims are the further investigation of the release characteristics of the liposomes and determination of the preferential accumulation area in tumor tissue, in order to establish a liposomal combination therapy for a safe and selective tumor treatment.
